# Randomised Controlled Study on Measures to Increase Vaccination Rates among German Armed Forces Soldiers

**DOI:** 10.3390/ijerph19148568

**Published:** 2022-07-14

**Authors:** Jana Nele Arnold, Nils Gundlach, Irina Böckelmann, Stefan Sammito

**Affiliations:** 1 Bundeswehr Hospital Hamburg, 20359 Hamburg, Germany; nelearnold@bundeswehr.org; 2 Department of Occupational Medicine, Otto-von-Guericke Universität, 39106 Magdeburg, Germany; irina.boeckelmann@med.ovgu.de; 3 Medical Clinic Rotenburg (Wümme), 27356 Rotenburg, Germany; nilsgundlach@bundeswehr.org; 4 German Air Force Centre for Aerospace Medicine, 51147 Cologne, Germany

**Keywords:** vaccinations, vaccination rates, prevention, military, infectious diseases

## Abstract

Vaccination is one of the most effective medical measures for preventing infectious diseases. Even though there are recommendations for specific occupational groups that have an increased risk of infection, e.g., armed forces personnel, there are gaps in the vaccination rates of this personal. We conducted a randomised and controlled cohort study to examine whether a computerised reminder system would increase the vaccination rates of active soldiers over a period of twelve months. A total of 506 soldiers with a mean age of 27.7 ± 6.5 years (experimental group (EG)) and 27.9 ± 6.3 years (control group (CG)) were included in our study. Only 26.2% of the EG and 31.3% of the CG had received the required vaccinations at the beginning of our study. The vaccination rates for influenza (50.5% and 49.1%) and tick-borne encephalitis (57.1% and 60.7%) were particularly low, for measles, mumps, and rubella they were high (94.3% and 97.8%). A highly significant increase (*p* < 0.001) in vaccination rates was observed for both groups during our study. The results revealed considerable vaccination gaps among German armed forces soldiers. Despite a highly significant increase in vaccination rates during the study, there is still a clear need for action.

## 1. Introduction

Vaccinations are among the most effective primary preventive medical measures. Since the end of the 18th century, various vaccinations have been developed and approved. This has drastically reduced incidences of potentially lethal diseases [1,2]. At the international level, the World Health Organization (WHO) issues vaccination recommendations [3]. At the national level, these recommendations are adapted to the hygienic, medical, and infectiological conditions in each country. The responsible agency in the Federal Republic of Germany is the Standing Committee on Vaccination (Ständige Impfkommission). It prepares and updates vaccination recommendations [4].

In Germany, childhood vaccination programmes have achieved a high primary vaccination rate for the twelve standard vaccinations. Nationwide vaccination rates are documented every year as part of school entry examinations in accordance with Section 34(11) of the German Infection Protection Act (Infektionsschutzgesetz). Data from 2017 show that, of 649,847 children, 93.8% were completely immunised against tetanus. Similar figures were noted for diphtheria (93.6%), pertussis (93.2%), and polio (92.9%). In comparison to data from school entry examinations in 2014, however, rates had dropped by 2.2 percentage points [5]. The large-scale German health survey for children and adolescents (KIGGS study) from 2003 to 2006 also showed high primary vaccination rates among 14,603 children. For these children between the ages of 2 and 17, the rates were 93.0% for tetanus, 92.6% for diphtheria, and 90.8% for polio. The survey found deficits, however, regarding booster shots. For example, only 57.0% of children received their first tetanus booster injection on time [6]. 

Studies with adult participants also revealed that vaccination coverage is not ideal. A large-scale study from Schleswig-Holstein, for example, showed that 76.5% of participants under 20 years of age were fully immunised against tetanus, diphtheria, and polio as opposed to only 32.9% of participants older than 60 years of age [7]. A study by Poethko-Müller et al. from 2013 showed similar results. Based on a total of 11,260 vaccination certificates, this study showed that 71.4% of the participants were currently immunised against tetanus and 57.1% against diphtheria. Here too, younger participants had better vaccination rates [8].

National vaccination recommendations also include recommendations for specific occupational groups and for certain diseases [4]. Soldiers in particular are exposed to special occupational infection risks [9]. Possible vaccinations for the German armed forces (Bundeswehr) are listed in Publication A1-840/8-4000 [10]. These guidelines closely follow the national recommendations of the Standing Committee on Vaccination, and every soldier has to have certain standard vaccinations. These include tetanus, diphtheria, polio, pertussis, measles, mumps, rubella (MMR), hepatitis A (Hep A), hepatitis B (Hep B), and influenza vaccinations. In addition, personnel for assistance and disaster relief operations at home must be vaccinated against tick-borne encephalitis (TBE). There are also specific vaccination programmes for each mission abroad which are adapted to the hygienic conditions and infectious diseases of the region in question. The vaccination status of soldiers is monitored by disciplinary superiors, agency heads, or a person authorised by them. This task does not require any special training or qualification. Soldiers receive vaccinations from the medical facility that is normally responsible for them [11]. 

On the whole, international scientific literature on vaccination rates for military personal is inadequate. A systematic review of the literature has shown, however, that around the world there are noticeable deficits regarding vaccination rates among this special occupational group [12]. No data have been published on the vaccination status of Bundeswehr personnel. Unit physicians have reported, however, that many soldiers are not properly vaccinated for assistance and disaster relief operations at home, which in turn may jeopardise operational readiness and deployability. 

The aim of this study is thus to examine vaccination coverage and the influence of individualised reminders on the behaviour of soldiers with regard to vaccination.

## 2. Materials and Methods

We conducted a randomised and controlled cohort intervention study with two groups (experimental (EG) and control groups (CG)) and collected data at five points over a twelve-month period from 2016 to 2017. 

The study population consisted of every active-duty soldier at Lent barracks in Rotenburg (Wümme), Lower Saxony, in northern Germany. At the time of the study, the barracks was home to Light Infantry Battalion 91 with its five companies, a staff, and a training support company as well as the 3rd company of Logistic Support Battalion 141 and a medical clinic (991 soldiers in total). Due to various factors (training, deployments abroad, leave, etc.) only 694 soldiers were present at the garrison at the beginning of the study. Initially, the study included all soldiers who volunteered to participate after having received written and signed information about the study and who submitted their vaccination certificates by the beginning of the study on 1 March 2016 (*n* = 588, response rate = 84.7%).

**Figure 1:**
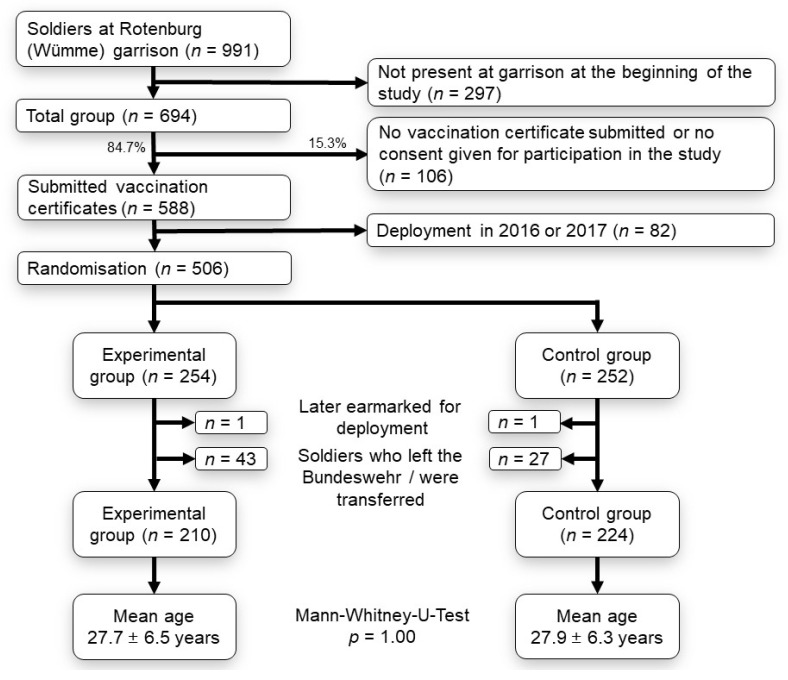
Flow chart on participant numbers (*n*) with exclusion criteria, subsequent randomisation and calculation of mean age.

First and last names, sex, date of birth, unit, and all planned deployments abroad for 2016 and 2017 were entered into a Microsoft Access database at the beginning of the study. All documented tetanus, diphtheria, polio, pertussis, hepatitis A, hepatitis B, TBE, MMR, and influenza vaccinations were transferred from each vaccination certificate to the database. In all cases where primary vaccination was transferred from an old vaccination certificate to a new certificate without exact vaccination data, we assumed that the primary vaccination was complete. On the basis of the corresponding recommendations [10], we ascertained separately for every vaccination whether and up to what date the participant would be or was immunised. Soldiers who, at the beginning of the study, were earmarked for a deployment abroad were excluded from further participation in the study (*n* = 82). The remaining participants (total number at the beginning of the study, *n* = 506) were then randomised by assigning them alternately to the EG or the CG. Figure 1 shows an overview with a flow chart of the participant numbers with exclusion criteria, subsequent randomisation, and calculation of mean age. In the course of the study, all vaccinations administered in the treatment room of the military medical clinic at Rotenburg (Wümme) were documented in the database, and the new date of full immunisation was added. 

For every single vaccination, we documented on 1 March 2016 (T0), 1 June 2016 (T1), 1 September 2016 (T2), 1 December 2016 (T3), and at the end of data collection on 1 March 2017 (T4) whether the soldiers were immunised against various diseases (yes/no). In addition, members of the EG received a personal reminder by mail at the time points T0–T3. Members of the CG were, however, reminded of necessary vaccinations by the person in charge of vaccination management, but they did not get a personal written invitation by mail from the database. 

Two soldiers who, in the course of the study, were earmarked for deployment abroad were excluded from further analysis regardless of their initial allocation. This was done because vaccinations are administered with greater urgency when a deployment abroad is planned. Furthermore, soldiers who were transferred within the armed forces during the study or who left the Bundeswehr were no longer taken into consideration (n = 70). In the end, 210 participants were in the EG and 224 in the CG. An intention-to-treat analysis was carried out, i.e., all soldiers that began the study were taken into consideration. Figure 1 provides an overview of the inclusion and exclusion criteria and the numbers and ages of participants.

After the data were anonymised, a statistical analysis was carried out using SPSS for Windows, version 24.0 (SPSS Inc., Chicago, IL, USA). Mean and standard deviation were calculated for the average age of all participants, and the independence of the groups was examined in a bivariate analysis with the chi-squared test. 

The vaccination rates at the various dates were calculated as percentages, and the absolute increase in percentage between T0 and T4 was calculated. This was followed by a test for independence of the groups by means of a bivariate analysis with the chi-squared test in cases where there were immunisations for the various diseases at T0. The same procedure was carried out for the presence of full immunisation for assistance and disaster relief operations at home. We then calculated the percentage of participants who had a certain number of full immunisations.

The Friedman test was also applied to determine whether vaccination rates had significantly increased from T0 to T4. This test was supplemented by the Wilcoxon test in order to determine at which points the differences were statistically significant. The significance level for all statistical tests was defined as *p* < 0.05. In order to adjust for multiple comparisons, a Bonferroni correction was used. 

The study was carried out in accordance with The Code of Ethics of the World Medical Association (Declaration of Helsinki) for experiments involving humans and was approved by the ethics committees of the Medicine University of Magdeburg (149/15) and by the research authorization of the German Military Academy (05KS-S-50 1617). There is no registration in a clinical trial because the regulation at the beginning of the study did not require registration in a national Clinical Trial Registry Platform for such a study.

## 3. Results

The average age of soldiers was 27.7 ± 6.5 years in the EG and 27.9 ± 6.3 years in the CG. There were no significant age differences between the two groups (*p* > 0.05). The number of females in both groups were similar (EG: 20 (7.9%), CG 17 (6.8%), *p* = 0.626). Initial immunisation at T0 was between 50.5% for influenza and 94.3% for MMR in the EG and between 49.1% for influenza and 97.8% for MMR in the CG (see Table 1).

The vaccination rates at T4 rose to values between 63.3% for influenza and 97.1% for MMR in the EG and between 64.7% for influenza and 99.1% for MMR in the CG. This corresponds to a total increase of 2.8% (MMR) to 20.0% (TBE) in the EG and of 1.3% (MMR) to 17.4% (TBE) in the CG.

The chi-squared test at the beginning of the study showed a significant group difference (*p* = 0.021) for polio; no significant group difference was found for the other vaccinations (*p* > 0.05).

At the beginning of the study, 26.2% of the EG and 31.3% of the CG were fully immunised for assistance and disaster relief operations at home; there was no significant difference between the groups (*p* > 0.05) (Table 1). The percentage of soldiers who were fully immunised for assistance and disaster relief operations at home increased after 12 months to 47.1% in the EG and 48.2% in the CG (Table 2).

**Figure 2:**
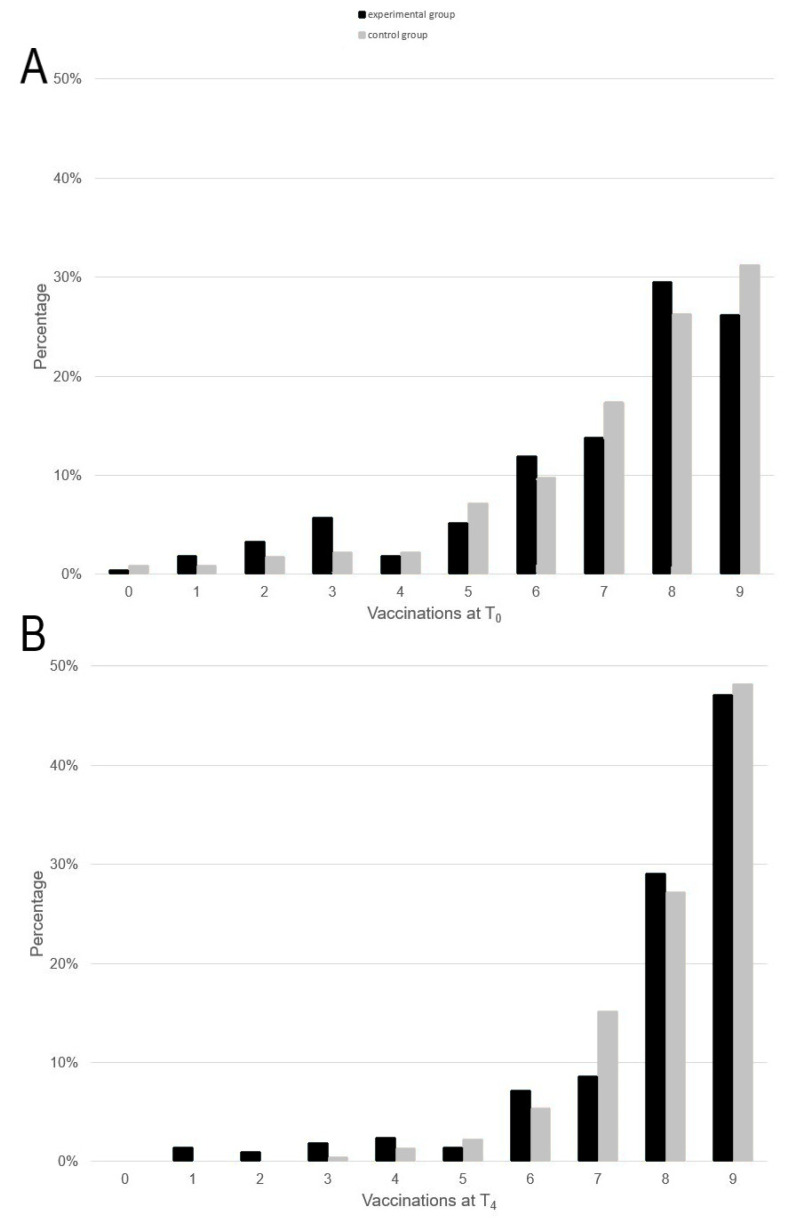
Development of full immunisation against the diseases applicable to assistance and disaster relief operations at home (tetanus, diphtheria, polio, pertussis, measles, mumps, rubella, hepatitis A, hepatitis B, tick-borne encephalitis, influenza) during the study period, X-axes show the number of full immunisations, maximum 9 (**A** = vaccinations at beginning of the study (T0), **B** = vaccinations at the end of the study (T4)).

The percentage of participants at T0 and T4 who are fully immunised against 0–9 diseases (based on the nine standard vaccinations) is shown in Figure 2. This figure shows that, for both groups at T0, less than 10% of the participants were not fully immunised against any infectious diseases. The same applies for one to five infectious diseases. About 10% of participants were fully immunised against six infectious diseases, about 15% against seven, about 25–30% against eight, and almost 30% of participants were fully immunised against all nine diseases at the start of the study. There was a clear shift of values towards the right by T4; neither group had any soldiers who were not immunised against any of the infectious diseases. In the CG, all participants were immunised against at least three diseases. The percentage of complete immunisation for all nine diseases (standard vaccinations) increased to almost 50% in both groups.

There is a highly significant increase in vaccination rates from T0 to T4 (*p* < 0.001). A Wilcoxon test was also carried out. It also showed significant changes over time (even after correction for multiple testing). Significance was above the corrected significance level of *p* < 0.0125 only in the CG from T0 to T3, when *p* = 0.077 (Table 3). It should be noted that the annual vaccination against influenza expired before T3 and had to be vaccinated again. Therefore, there was a selective change in vaccination rates.

Between the two groups there was no significant difference (*p* > 0.05) at all time points (T0–T4) in regard to the number of vaccinations overall nor if they had a vaccination status against all nine immunisations that were recommended (Table 4).

The intention-to-treat analysis, which also considered the soldiers who had left the study, also showed a significant increase in full immunisation for all vaccinations examined (data not presented). Here as well, no significant difference between the two groups was found at the beginning of the study with regard to complete immunisation for assistance and disaster relief operations at home.

## 4. Discussion

Our study showed that there are considerable vaccination gaps among Bundeswehr soldiers. Initially, only every fourth soldier had complete immunisation for assistance and disaster relief operations at home. 

The vaccination rates for Hep A, Hep B, TBE, and influenza were well below the desired levels. None of these vaccinations are among the standard vaccinations recommended by the Standing Committee on Vaccination for adults. Hep A is an extra vaccination for at-risk people, e.g., for health care personnel or people travelling to destinations with a high prevalence of Hep A [4]. The reason why the vaccination rate is relatively high compared to TBE and influenza is that only two vaccinations are necessary to achieve full immunisation and that protection lasts for 25 years [10]. Of the vaccinations mentioned above, Hep B is characterised by a relatively high vaccination rate, which is likely because it is a standard childhood vaccine. Our study group has an average age of 27 years and is thus very young. Our test persons likely had partial immunisation when they entered the Bundeswehr, as Hep B booster shots are recommended up to age 17 [4]. This assumption was confirmed in a cross-sectional study in which it was shown that recruits to the Bundeswehr had a vaccination rate of 16.6% (unpublished data). In addition, after primary immunisation in childhood is achieved, a one-time booster shot is sufficient to restore immunisation [4]. The vaccination rate for TBE was very low, which is likely due to the fact that TBE is endemic primarily in southern Germany [13]. The study group was based in northern Germany, however, and thus there was no indication for such a vaccination in the civilian sector [4]. Additionally, a cross-sectional study at the garrison showed that only 2.4% of recruits were fully immunised when they joined the military (unpublished data). This is why major efforts are necessary after recruitment to ensure a satisfactory rate of full immunisation. The lowest vaccination rate was for influenza. This can be partly explained by the fact that, in the civilian sector, influenza vaccinations are an extra vaccination for at-risk people and would thus generally not be indicated for this young cohort outside the Bundeswehr. In addition, immunisation against influenza must be refreshed annually [4]. This high vaccination frequency means that influenza is an especially sensitive parameter for good vaccination compliance. Influenza vaccinations from the 2015/2016 season were no longer effective as of 1 October 2016. More soldiers were, however, vaccinated against influenza at T3 than at T0 (start of study). This shows that the reminder system had increased awareness of vaccination among the participating soldiers. 

The highest vaccination rate was for MMR because this vaccination guarantees lifelong immunisation after two doses in childhood or a one-time application for adults [10]. Since the rates for vaccinations that require frequent booster shots are significantly lower than the rates for those that rarely require booster shots, the vaccination gaps we found appear to be caused less by a lack of primary immunisation and more as a result of missing booster shots. 

The large-scale DEGS1 study by Poethko-Müller et al. found a current immunisation rate of 75.6% for tetanus, 68.6% for diphtheria, and 28.4% for pertussis for the 18–29-year age group in the civilian sector. For the other examined vaccinations, the study merely noted whether at least one vaccination against the disease had been administered; this is why the studies are not comparable [8]. A study by Bader et al. provided similar results; for the age group of 20–29 years, it identified a current immunisation rate of 73.6% against tetanus, 68.5% against diphtheria, and 37.6% against MMR (at least a one-dose vaccination) [7]. On the whole, these rates are significantly lower than all the vaccination rates we identified in our study; for this reason, the vaccination monitoring carried out by superiors or persons responsible for vaccination management appears to be effective.

A comparison with other armed forces shows that the Bundeswehr has a slightly lower vaccination rate for tetanus [14,15,16], similarly high vaccination rates for polio and Hep B [14,17,18], and considerably higher vaccination rates for all other examined vaccinations [14,19,20]. This indicates that, in general, vaccination monitoring works in the Bundeswehr although there is clearly need for improvement.

A possible reason why vaccinations were not carried out is incorrect or inadequate vaccination monitoring, which is often carried out by people with no medical training. In addition to the approach chosen in this study, namely electronically preparing and mailing reminders, a possible solution would be to conduct training courses for unit personnel in charge of vaccination management to ensure a common level of knowledge. Another possible reason for vaccination gaps is poor awareness on the part of soldiers. For example, a study carried out by the armed forces of Saudi Arabia showed that 50.3% of the surveyed soldiers did not know whether they were currently immunised against influenza [21]. A possible approach to solving this problem would be to provide soldiers with more information (e.g., in briefings or one-on-one talks) in order to increase their initiative to independently consult a medical officer for necessary booster vaccinations. In our study, however, for practical reasons we decided in favour of automatically prepared and mailed reminders. We did this to rule out the possible confounding role of the personal and individual capabilities of personnel in charge of vaccination management. Since the reminders are electronically prepared, it required little effort to repeatedly inform all participants.

Vaccination gaps were significantly reduced in the course of our study. At the end of the study, 50% of the participating soldiers were sufficiently immunised. For all vaccinations apart from influenza and TBE, the achieved vaccination rates were around 90%, which is a good result. We found that many soldiers needed only one or two vaccinations to achieve full immunisation. This leads us to believe that soldiers are willing to be vaccinated. However, the vaccination rates for influenza (63.3% and 64.7%) and TBE (77.1% and 78.1%) are especially insufficient and need to be improved. 

Furthermore, individual soldiers may have a negative attitude towards vaccination despite the fact that compulsory vaccination is ordered by directive for this occupational group. In a study on 942 soldiers of the Israeli Air Force, 10.8% said that they had actively refused a vaccination [19]. Of these soldiers, 37.7% were concerned about adverse effects, 32.1% did not believe in the effectiveness of vaccination, and 23.1% rejected vaccination on principle. The data at our disposal cannot be used to answer the question as to what extent some of the military personnel examined here rejected vaccination on principle and therefore, despite the intervention and personalised cover letter, did not achieve full immunisation within twelve months. 

We initially assumed that an automated written personal reminder delivered on a regular basis would improve vaccination rates compared to previous procedures. Unfortunately, this was not demonstrated because vaccination rates increased equally in both groups. Although initially randomised, the two groups were able to communicate with each other as the soldiers were stationed at the same base. This may have increased the awareness of vaccination in the CG, which in turn may have led to a database bias. Regrettably, we were unable to involve random participants from two independent locations since it was impossible in this study to create comparable conditions (e.g., operational options and composition of the stationed units). The vaccination rates of only 26% and 31% at the beginning of the study show that the current standard procedure involving persons tasked with vaccination management is inadequate for achieving a sufficiently high vaccination rate. As this procedure has been established in the Bundeswehr for several years, we can assume, at least in a pretest-posttest comparison, that the reminder system increased awareness of vaccination among the subjects.

From our perspective, the best way for future intervention studies in this area should be the inclusion of soldiers with similar general conditions from several locations to make sure that the two groups are not able to influence each other. A second approach could be to observe a group of soldiers over an initial study period without intervention (e.g., one year) and then to analyse the effect of the intervention in a second year. A general vaccination registry for all soldiers (not implemented at this moment) with the possibility to analyse all information without data collecting would be a great step forward to improve the vaccination status of the entire armed forces.

The strengths of our study were the large number of participants (n = 506) as well as the response rate (84.7%). The results can thus be regarded as representative for the chosen location. Randomisation took place but was, however, inadequate because communication between the groups apparently led to a bias. In addition, the participants were not randomised according their vaccination status with the effect that the baselines of the two groups were not directly the same. Even the difference was not significant, it could have had an impact on the results. In a military setting with a closed population, however, this leads to increased effectiveness because it also raised awareness of vaccination among soldiers who had not received a reminder. Further studies could compare soldiers at different locations in order to improve the quality of data.

## 5. Conclusions

There are considerable vaccination gaps among Bundeswehr soldiers. Despite the considerable increase in full immunisation rates, there are nevertheless deficits in the fulfilment of requirements. This shows that a multimodal approach is necessary in order to achieve this goal. The electronic vaccination status monitoring system examined here, which includes automated reminders, appears to be an effective instrument to close these gaps.

## Figures and Tables

**Table 1:** Vaccination rates (absolute numbers) of the experimental (EG) and control groups (CG) at T0 and the results of the bivariate analysis by means of the chi-squared test. Overall protection is defined as a full vaccination status against all nine immunisations listed above. MMR = measles, mumps, rubella, TBE = tick-borne encephalitis.

	Vaccination Rate EG (n = 210)	Vaccination Rate CG (n = 224)	Chi^2^ Test (2-Sided)
Tetanus	84.8% (178)	91.1% (204)	0.054
Diphtheria	85.2% (179)	91.1% (204)	0.073
Polio	81.4% (171)	89.3% (200)	**0.020**
Pertussis	88.6% (186)	87.5% (196)	0.769
MMR	94.3% (198)	97.8% (219)	0.082
Hepatitis A	80.5% (169)	83.0% (186)	0.535
Hepatitis B	77.1% (162)	79.0% (177)	0.644
TBE	57.1% (120)	60.7% (136)	0.494
Influenza	50.5% (106)	49.1% (110)	0.848
Overall protection	26.2% (55)	31.3% (70)	0.289

**Table 2:** Vaccination rates (absolute numbers) of the experimental (EG) and control groups (CG) at T0 and T4 as well as absolute increases in vaccination rates during the study. Overall protection is defined as a full vaccination status against all nine immunisations listed above. MMR = measles, mumps, rubella, TBE = tick-borne encephalitis.

	Vaccination Rate EG			Vaccination Rate CG		
	T0	T4	Δ (T0–T4)	T0	T4	Δ (T0–T4)
Tetanus	84.8% (178)	90.5% (190)	+5.7%	91.1% (204)	96.0% (215)	+4.9%
Diphtheria	85.2% (179)	90.5% (190)	+5.3%	91.1% (204)	96.0% (215)	+4.9%
Polio	81.4% (171)	88.1% (185)	+6.7%	89.3% (200)	95.1% (213)	+5.8%
Pertussis	88.6% (186)	95.7% (201)	+7.1%	87.5% (196)	92.0% (206)	+4.5%
MMR	94.3% (198)	97.1% (204)	+2.8%	97.8% (219)	99.1% (222)	+1.3%
Hepatitis A	80.5% (169)	92.4% (194)	+11.9%	83.0% (186)	95.5% (214)	+12.5%
Hepatitis B	77.1% (162)	90.5% (190)	+13.4%	79.0% (177)	91.5% (205)	+12.5%
TBE	57.1% (120)	77.1% (162)	+20.0%	60.7% (136)	78.1% (175)	+17.4%
Influenza	50.5% (106)	63.3% (133)	+12.8%	49.1% (110)	64.7% (145)	+15.6%
Overall protection	26.2% (55)	47.1% (99)	+20.9%	31.3% (70)	48.2% (108)	+16.9%

**Table 3:** Results of the Friedman test for significant changes in vaccination rates from T0 to T4 and the Wilcoxon tests between the various time points.

	pFriedman T1, T2, T3 and T4	pWilcoxon T0–T1	pWilcoxon T0–T2	pWilcoxon T0–T3	pWilcoxon T0–T4
**Comparison of vaccination rates at various times in the experimental group (*n* = 210)**	**<0.001**	**<0.001**	**<0.001**	**0.002**	**<0.001**
**Comparison of vaccination rates at various times (2-sided) in the control group (*n* = 224)**	**<0.001**	**0.005**	**0.002**	0.077	**<0.001**

**Table 4:** Results of the chi^2^-test for significant changes between the two groups for T0–T4.

	T0	T1	T2	T3	T4
**Comparison experimental group vs. control group (over all** **vaccinations)**	0.479	0.979	0.387	0.233	0.104
**Comparison experimental group vs. control group (only full vaccinated vs. non-full-** **vaccinated)**	0.124	0.572	0.568	0.874	0.662

## Data Availability

The data that support the findings of this study are available from the Federal Ministry of Defense. Data are available on reasonable request.
